# Prevalence and associated factors of baseline anemia among cervical cancer patients in Tikur Anbesa Specialized Hospital, Ethiopia

**DOI:** 10.1186/s12905-021-01185-9

**Published:** 2021-01-25

**Authors:** Mulugeta Wassie, Agazhe Aemro, Beletech Fentie

**Affiliations:** School of Nursing, College of Medicine and Health Sciences, University of Gondar, Gondar, Ethiopia

**Keywords:** Cervical cancer, Anemia, Ethiopia, Prevalence

## Abstract

**Background:**

Almost one patient with cancer in two is anemic. About 40 to 64% of cervical cancer patients are anemic at time of presentation. The rate of anemia increases with the use of chemotherapy, radiotherapy, hormonal therapy and associated with poorer treatment outcome and quality of life. Therefore, the aim of this study was to assess prevalence and associated factors of baseline anemia among cervical cancer patients in Tikur Anbesa Specialized Hospital (TASH), Ethiopia.

**Methods:**

Institutional based cross-sectional study was done from March to April 2019 at TASH cancer center. Data were collected from patient’s chart using structured checklist and analyzed using Stata14.2. Binary logistic regression model was used to identify covariates which affected the outcome variable.

**Result:**

This is a 3-years retrospective study from 2014 to 2016. The prevalence of baseline anemia among cervical cancer patients was 50.95%. Being stage IV [AOR = 2.38, 95% CI (1.21–4.67)], having comorbidity [AOR = 3.32, 95% CI (2.25–4.90)] and using substances (patients who used one, two or all of the three substances (cigarate, chat and alcohol)) [AOR = 2.03, 95% CI (1.21–3.41)] significantly increased the occurrence of anemia while being divorced [AOR = 0.6, 95% CI (0.36–0.98)] decreased the occurrence of anemia in the current study.

**Conclusion:**

The prevalence of baseline anemia was high in the current study compared to other literatures. Significant factors of baseline anemia of cervical cancer in the current study were advanced stage (stage IV), presence of comorbidity, substance usage and being divorced (protective). The authors recommend that it is better to give special attention to those patients with the stated factors that could interfere treatment outcome.

## Background

The world cancer burden is estimated to have risen to 18.1 million new cases and 9.6 million deaths in 2018. One in five men and one in six women in the globe develop cancer during their life, and one in 8 men and one in 11 women die from the disease. Worldwide, the total number of people who are alive within 5 years of a cancer diagnosis called the 5-year survival is estimated to be 43.8 million. Cancer of cervix ranks fourth for both incidence (6.6%) and mortality (7.5%) [1].

Anemia of chronic disease, the second most prevalent anemia, is caused by iron deficiency which happens in patients with acute or chronic immune activation. It occurs as a result of cytokines and cells of the reticuloendothelial system make changes in iron homeostasis. Fatigue has larger harmful influence on cancer patients’ daily lives than many other cancer treatment related complications. Cancer patients with anemia are also facing many emotional and mental costs in addition to lack of self-motivation, sorrow, frustration and mental collapse [2–4].

Almost one patient with malignancy in two is anemic, with the consequence of poorer treatment outcome and quality of life. A low hemoglobin (Hb) level seems to directly affect treatment efficacy, predominantly of radiotherapy, probably due to low tissue oxygenation causing decrease in radio-sensitivity. Tissue hypoxia might also enable tumor progression and impair the efficacy of cytostatic drugs [5].

About 40–64% of cervical cancer patients presenting for cancer therapy were anemic and the rate rise with the use of chemotherapy, radiotherapy and hormonal therapy in the research conducted in Australia. It may result in a significant reduction in oxygen delivery to tumors, but compensatory mechanisms reduce the impact on tumor oxygenation which can be connected with poorer treatment outcome in cervical cancer [6].

Anemia in cervical cancer patients can be due to tumor-related bleeding, tumor invasion of bone marrow, malnutrition caused by the tumor, abnormal iron metabolism, renal physiology impairment and compromised bone marrow function [6, 7]. Hb level below 10 g/dl during radiation therapy of cervical cancer was significant and it was correlated with lower survival of the patients. Similarly low Hb level before chemotherapy significantly lowered the survival of cervical cancer patients [8, 9].

Patients who completed a full course of radiation treatment for advanced cancer of cervix showed that pretreatment Hb and its level during treatment affected the prognosis negatively. Patients who had at least one Hb value below10 g/dl during treatment had twice the risk of loco regional failure compared with patients whose Hb levels were 10 g/dl [10, 11].

A study conducted in TASH on the prevalence and associated factors of anemia among patients with solid tumor reveled that the overall prevalence of anemia across different tumor was 23% and higher (37.7%) prevalence was observed in gynecological cancers. Similarly, the study conducted by the European Cancer Anemia Survey (ECAS) showed that prevalence of anemia at admission was 39.3% and performance status and bleeding history were significantly associated for occurrence of anemia [12, 13]. Therefore, the aim of this study was to assess the prevalence and associated factors of baseline anemia among cervical cancer patients in TASH, Ethiopia.

## Methods

### Study design, period and area

Institutional based cross-sectional study was done at the cancer center of TASH from March to April 2019. TASH is the biggest referral public hospital in Ethiopia established in 1972. It is the training center of health professionals including undergraduate and postgraduate medical students, dentists, nurses, pharmacists, laboratory technicians and others paramedics.

The hospital is staffed by many health professionals from various disciplines. It has total beds of 800 and the beds reserved for cancer care are 20. TASH is the only center providing comprehensive cancer care in Ethiopia.

### Populations

All medical records of women diagnosed with cancer of the cervix in TASH cancer center were source populations and all medical records of cancer of the cervix who attended in cancer center of TASH from January 1, 2014 to December 31, 2016 were study populations. Cervical cancer patients with incomplete medical charts and charts that were not found during data collection period were excluded.

### Sample size determination, sampling procedure and study variables

All patients with cancer of the cervix diagnosed at TASH cancer center from January 2014 to December 2016 was the total sample size. Census sampling procedure was used and the procedure was as follows: at the beginning, profiles of all women with cancer of the cervix who diagnosed between January 2014 to December 2016 in the TASH was assessed and 1227 cervical cancer patient charts were found. 634 charts that fulfilled the inclusion criteria (charts with complete information) were identified and finally data were collected from 634 patient medical records. Baseline anemia was the outcome variable and Sociodemographic characteristics (marital status, residential address, age at diagnosis, substance use, number of children, region, occupation, religion), pathological and clinical factors (stage at presentation, histology type, comorbidity and types of comorbidity) were independent variables.

### Operational definitions

Anemia: patients’ Hb level below 12.0 g/dl was classified as anemic [14, 15]

Comorbidity: The presence of any conditions (mentioned in the Carlson comorbidity Index [16]) other than cervical cancer at diagnosis which was designated as “yes” in the checklist.

Substance use: Patients who used one, two or all of the three substances (cigarate, chat and alcohol) [17].

Stage at diagnosis: The revised FIGO staging for carcinoma of the vulva, cervix, and endometrium was used in this study [18].

### Data collection instrument, quality assurance and collection procedures

Data were collected from patients’ charts using pre-tested and structured checklist prepared in English. The checklist consisted of two parts: (1) sociodemographic and individual level factors and (2) pathological and clinical factors. Two supervisors having second degree in oncology nursing and three data collectors having first degree in nursing were involved in the data collection process. One day intensive training was given to data collectors and supervisors. Pretest was conducted by considering 5% of the total sample size to test its consistency with actual data collection in the charts recorded during the study period and necessary corrections were done accordingly. Every day after data collection, checklists were reviewed and checked for completeness, accuracy and clarity by the supervisors and principal investigator and the necessary feedback was offered to data collectors.

### Data processing and analysis

Data were coded and then entered, edited and cleaned using Epi-data 3.1 and exported to STATA14.2 statistical software for analysis. Frequencies, proportions and descriptive statistics were used to describe the study population in relation to relevant variables and was presented using tables and graphs. Binary logistic regression model was used to analyze factors that affect the outcome variable.

Before running the Binary logistic regression model, assumption of the model fitness was checked. The model assumption was checked using Hosmer and Lemeshow test and the test result with *P* value > 0.05 indicates the data fulfilled the model assumption [19]. The *P* vale of Hosmer and Lemeshow test was 0.129 in this study indicating the data fitted the binary logistic regression model. Variables in multivariable binary logistic regression with a *P* value < 0.05 were considered to have actual interference with the outcome variable(anemia) with 95% confidence interval.

### Ethical clearance

Ethical approval for this study was obtained from the Institutional Review Boards of school of nursing and midwifery, Addis Ababa University. The letter of permission was written from school of nursing and midwifery to the cancer center of TASH. Then, the cancer center chief administrator allowed to collect the data from the cervical cancer patents medical records. The study was conducted without individual informed consent since it relies on chart review.

## Results

### Socio-demographic characteristics of cervical cancer patients

The study was conducted within 634 cervical cancer patients attending in TASH cancer center. The mean age of study patients was 49.82 years with SD ± 11.72. More than half (n = 355) of the patients came from urban area and 203(32%) of came from Oromia regional state of Ethiopia. Orthodox religion followers accounted 58.04%. About 42% were house wives and nearly one sixth (n = 106) were substance users. Approximately two third (n = 407) were married and 42% of the study patients have more than three children (Table 1).

**Table 1:** Socio demographic characteristics of cervical cancer patients in TASH cancer center, Ethiopia (n = 634)

Variables	Code	Baseline anemia		Total N (%)
		Yes N (%)	No N (%)	
Age	< 30	30(50.85)	29(49.15)	59(9.30)
	30–39	44(36.97)	75(63.02)	119(18.77)
	40–49	95(57.92)	69(42.07)	164(25.9)
	50–59	87(56.13)	68(43.87)	155(24.45)
	≥ 60	67(48.91)	70(51.09)	137(21.61)
Residence	Urban	184(51.83)	171(48.17)	355(56.00)
	Rural	139(49.82)	140(50.18)	279(44.00)
Region	Amhara	83(51.55)	78(48.45)	161(25.39)
	Oromia	103(50.74)	100(49.26)	203(32.02)
	Tigray	18(78.26)	5(21.74)	23(3.62)
	SNNP	32(53.33)	28(46.67)	60(9.46)
	Addis Ababa	77(46.11)	90(53.89)	167(26.34)
	Others	10(50.00)	10(50.00)	20(3.16)
Religion	Orthodox	176(47.82)	192(52.17)	336(58.04)
	Muslim	70(23.49)	69(20.54)	139(21.92)
	Protestant	49(16.44)	64(19.05)	113(17.82)
	Others	3(1.01)	11(3.27)	14(2.21)
Occupation	Gov’t employee	42(49.41)	43(50.59)	85(13.41)
	Merchant	40(54.79)	33(45.20)	73(11.51)
	Farmer	60(51.28)	57(48.71)	117(18.45)
	House wife	133(49.44)	136(50.56)	269(42.43)
	Others	48(53.33)	42(46.67)	90(14.19)
Substance use	User	73(68.86)	33(31.13)	106(16.71)
	None user	250(47.35)	278(52.65)	528(83.28)
Children	No child	8(40.00)	12(60.00)	20(3.15)
	One	20(48.78)	21(51.22)	41(6.46)
	Two	42(45.65)	50(54.35)	92(14.51)
	Three	115(53.49)	100(46.51)	215(33.91)
	More than 3	138(51.88)	128(48.12)	266(41.96)
Marital status	Married	218(53.56)	189(46.44)	407(64.20)
	Single	13(41.94)	18(58.06)	31(4.90)
	Widowed	48(48.98)	50(51.02)	98(15.46)
	Divorced	44(44.89)	54(55.10)	98(15.46)

Others: private employee, student, daily laborer

### Clinical and histopathological characteristics of cervical cancer patients

About two third (n = 413) of study patients presented at advanced stages (stage III and stage IV). Most (90.5%) cervical cancer patients had squamous cell carcinoma. Nearly one third (33.4%) of cervical cancer patients had comorbidity in which HIV accounted the majority (54.7%) of them (Table 2).

**Table 2:** Clinical and histopathological characteristics of cervical cancer patients in TASH cancer center (n = 634)

Variables	Code	Baseline anemia		Total N (%)
		Yes N (%)	No N (%)	
Stage	Stage I	26(41.26)	37(58.73)	63(9.93)
	Stage II	70(44.31)	88(55.69)	158(24.92)
	Stage III	118(46.27)	137(53.72)	255(40.22)
	Stage IV	109(68.99)	49(31.01)	158(24.92)
Histopathology	Squamous cell	299(52.09)	275(47.91)	574(90.54)
	Adenocarcinoma	24(40.00)	36(60.00)	60(9.46)
Comorbidity	Yes	152(71.70)	60(28.30)	212(33.44)
	No	171(40.52)	251(59.48)	422(66.56)
Types of comorbidity (n = 212)	HIV	90(76.27)	28(23.73)	118(55.66)
	Hypertension	31(63.27)	18(36.73)	49(23.11)
	Others	21(44.68)	16(34.04)	47(22.17)

### Prevalence of baseline anemia among cervical cancer patients

The prevalence of baseline anemia among cervical cancer patients was 50.95% (95% CI; 47.3–54.7). About 58% (n = 95) of anemic patients were with 40–49 age categories and nearly 52% (n = 184) of patients with anemia were urban dwellers. One hundred and seventy-six (47.82%) of anemia prevalence was observed among orthodox religion followers. Considering the patients regional state, approximately 78% (n = 18) of anemic patients came from Tigray region followed by SNNP (53%) and related to their occupation, merchants were most (54.79%) affected. The majority (n = 73) of substance users were anemic, about 54% (n = 218) of patients who are married were also affected with anemia. About 69% (n = 109) of stage IV, 52% (n = 299) with squamous cell carcinoma and 71.7% (n = 152) with comorbidity were anemic (Table 3).

**Table 3:** Prevalence of baseline anemia related to different characteristics of cervical cancer patients in TASH cancer center, Ethiopia (n = 634)

Variables	Code	Baseline anemia			
		Yes		No	
		Frequency	(%)	Frequency	(%)
Age	< 30	30	50.85	29	49.15
	30–39	44	36.97	75	63.02
	40–49	95	57.92	69	42.07
	50–59	87	56.13	68	43.87
	≥ 60	67	48.91	70	51.09
Residence	Urban	184	51.83	171	48.17
	Rural	139	49.82	140	50.18
Region	Amhara	83	51.55	78	48.45
	Oromia	103	50.74	100	49.26
	Tigray	18	78.26)	5	21.74
	SNNP	32	53.33	28	46.67
	Addis Ababa	77	46.11	90	53.89
	Others	10	50.00	10	50.00
Occupation	Gov’t employee	42	49.41	43	50.59
	Merchant	40	54.79	33	45.20
	Farmer	60	51.28	57	48.71
	House wife	133	49.44	136	50.56
	Others	48	53.33	42	46.67
Substance use	User	73	68.86	33	31.13
	None user	250	47.35	278	52.65
Children	No child	8	40.00	12	60.00
	One	20	48.78	21	51.22
	Two	42	45.65	50	54.35
	Three	115	53.49	100	46.51
	More than 3	138	51.88	128	48.12
Marital status	Married	218	53.56	189	46.44
	Single	13	41.94	18	58.06
	Widowed	48	48.98	50	51.02
	Divorced	44	44.89	54	55.10
Stage	Stage I	26	41.26	37	58.73
	Stage II	70	44.31	88	55.69
	Stage III	118	46.27	137	53.72
	Stage IV	109	68.99	49	31.01
Histopathology	Squamous cell	299	52.09	275	47.91
	Adenocarcinoma	24	40.00	36	60.00
Comorbidity	Yes	152	71.70)	60	28.30
	No	171	40.52	251	59.48
Comorbidity types (n = 212)	HIV	90	76.27	28	23.73
	Hypertension	31	63.27	18	36.73
	Others	21	44.68	16	34.04

### Factors associated with baseline anemia among patients with cancer of cervix

Independent variables were analyzed individually with the outcome variable and the variables with the *P* < 0.2 were included in the multivariable binary logistic regression model. In multivariable analysis, substance use, stage of cancer, comorbidity and marital status were significantly associated to the outcome variable at *P* value < 0.05 with 95% CI.

Cervical cancer patients with advanced stage (stages IV) presented with anemia 2.38 times [AOR = 2.38, 95% CI (1.21–4.67)] more than those patients with stage I.

Patients who used substances came with anemia about 2 times [AOR = 2.03, 95% CI (1.21–3.41)] more than those patients who were not substance users. Similarly, patients who had comorbidity presented with anemia 3.32 times [AOR = 3.32, 95% CI (2.25–4.90)] more than those who hadn’t comorbidity. Patients who had divorced were about 40% [AOR = 0.6, 95% CI (0.36–0.98)] less to be affected with anemia than those patients who were married (Table 4).

**Table 4:** Results of bivariable and multivariable binary logistic regression analysis of cervical cancer patients at Tikur Anbesa Specialized Hospital, Ethiopia (n = 634)

Variables	Code	COR (95% CI)	*P*-value	AOR (95% CI)	*P*-value
Age		1.04(0.53–2.05)	0.140*	1.01(0.99–1.02)	0.494
Residence	Rural	1.01(0.74–1.39)	0.110*	0.76(0.49–1.16)	0.204
	Urban	1		1	
Region	Amhara	1.4(0.59—3.32)	0.440	1.35(0.46–3.91)	0.577
	Oromia	1.59(0.68–3.70)	0.283	1.37(0.48–3.91)	0.552
	Tigray	6.50(1.47–28.80)	0.014*	3.78(0.87–16.23)	0.074
	SNNP	1.36(0.53–3.49)	0.524	1.57(0.49–4.95)	0.439
	Addis Ababa	0.91(0.39–2.17)	0.849	0.83(0.29–2.39)	0.737
	Others	1		1	
Occupation	Gov’t employee	1		1	
	Merchant	0.72(0.39–1.31)	0.182*	1.28(0.63–2.61)	0.481
	Farmer	0.80(0.46–1.39)	0.446	1.11(0.57–2.15)	0.754
	House wife	0.98(0.60–1.58)	0.921	1.16(0.65–2.06)	0.604
	Others	1.08(0.62–1.88)	0.778	1.32(0.66–2.59)	0.427
Number of children	No Child	1		1	
	One	2.23(0.84–5.92)	0.107*	1.01(0.29–3.51)	0.993
	Two	1.37(0.56–3.31)	0.488	1.00(0.31–3.21)	0.998
	Three	2.63(1.17–5.96)	0.020*	1.22(0.39–3.75)	0.730
	More than three	2.32(1.02–5.25)	0.044*	1.35(0.44–4.07)	0.600
Marital status	Married	1		1	
	Single	0.42(0.20–0.88)	0.020*	0.49(0.19–1.25)	0.136
	Widowed	0.56(0.34–0.92)	0.023*	0.86(0.52–1.40)	0.552
	Divorced	0.80(0.53–1.20)	0.285	0.60(0.36–0.98)	0.042**
Comorbidity	Yes	5.52(3.87–7.89)	0.001*	3.32(2.25–4.90)	< 0.001**
	No	1		1	
Histopathology	Adenocarcinoma	0.48(0.27–0.83)	0.009*	0.65(0.36–1.19)	0.170
	Squamous cell	1		1	
Stage	Stage I	1		1	
	Stage II	4.38(2.05–9.33)	< 0.001*	0.95(0.47–1.88)	0.876
	Stage III	2.62(1.26–5.44)	0.010*	1.15(0.61–2.14)	0.665
	Stage IV	10.29(4.82–21.99)	< 0.001*	2.38(1.21–4.67)	0.012**
Substance	User	3.96(2.59–6.04)	< 0.001*	2.03(1.21–3.41)	0.007**
	None user	1		1	

N.B: *variables associated with the outcome variable in bivariable analysis; **variables significantly associated with the outcome variable in multivariable analysis at 95% level of significant (*P* < 0.05); 1 = Reference category

## Discussion

This study aimed to assess the prevalence and associated factors of baseline anemia among cervical cancer patients in TASH, Ethiopia. Stage of the disease, comorbidity, substance use and marital status were significant factors that influenced the development of anemia among cervical cancer patients in the current study.

About 51% (95% CI 47.3–54.7) of cervical cancer patients in this study presented with baseline anemia. The current anemia prevalence is greater than the studies conducted in Canada [4], Korea [20], another study in Korea [7], Ethiopia [10] and Poland [21] with baseline anemia prevalence of 40%, 36.45, 12.3%, 37.7% and 33% respectively, but it is lower than the study conducted in New York [22] with 75% prevalence of anemia. The current anemic prevalence is inline with the study conducted in India with the prevalence of 54.7% [23]. This significance difference will be due to study time and sample size variation for all countries mentioned above and study setting, healthy care delivery policy differences and the countries priority to cancer treatment and prevention in case of Canada, Korea, Poland and New York.

Patients who presented with advanced stage (stage IV) had anemia about twofold more than those with stage I in the current study. This result is inline with the studies investigated in Canada(4), Korea [20], Poland [21] and Japan [24]. This might be due to the fact that as the stage of cancer get advanced, there could be cancer related bleeding in which this bleeding may cause blood loss anemia [7]. An other reason could be as the stage get advanced, load of cancerous cells will increase and at the same time the competition of nutrients could be also increased. This competition will decrease hemoglobin level of the patients and consequently lead to anemia. There will be also new blood vessel formation called angiogenesis. This angiogenesis process demands red blood cells that could lead to anemia [25, 26].

Substance users were also about two times more affected with anemia than none users in this study. This might be as a result of chemicals in substances (nicotin, hydrogen cyanide, lead, arsenic and benzene in cigarate [27]) decrease the production of red blood cells in the bone marrow, affecting the kidney which is the home of the production of erythropoietin (the hormone used for red cell production and maturation) [28–30].

An other evidence of this high prevalence of anemia among substance users could be as cigarette smoking largely affects hematopoiesis in a negative way. The level of oxyhemoglobin and the rate of delivery of oxygen to the tissues are the fundamental regulators of erythropoiesis. Decreased levels of erythropoietin in smokers compared with nonsmokers were found and suggested that the production may be downregulated by an elevated red-cell volume as positive feedback mechanism [29].

An other contributing factor of anemia in this study was commorbidity. Study patients with comorbidity were about 3.3 times more affected with anemia than those with none comorbid patients. This could be as a result of blood loss, low production of blood cells, decreased oxygen saturation and competition of red blood cells with diseases in addition to cancer [31, 32].

Patients who had divorced were 40% less to get anemia than those patients who were married in the current study. This might be related to pregnancy and give birth. The probability of getting pregnant and giving birth could be more in married patients than divorced and these conditions could increase the chance of acquiring anemia in married patients. But we couldn’t get other literatures that support the current study.

## Limitations and strength of the study

As a limitation, the data was collected from patient charts that might cause errors of recording since the data was not recorded for the purpose of research. Moreover, the data were collected on patients diagnosed from January 2014 to December 2016 that may not reflect the current situations. In addition, the study was conducted only in one cancer center in Ethiopia that will not represent the whole baseline anemia condition in the country. As a strength, the data were collected by nurses who were trained on cancer care that could increase the quality of the data and somehow big sample size that could increase the quality of statistics which would increase accuracy of inference to the general population.

## Conclusion

The prevalence of baseline anemia was high compared to other literatures in this study. Significant factors of baseline anemia of cervical cancer were; advanced stage (stage IV), presence of comorbidity, substance usage and being divorced (protective). The authors recommend that it is better to give special attention to those who have comorbidity at the time of diagnosis, patients with advanced stage and those patients who used substance since they could have anemia that interferes treatment and prognosis. In addition, it is better to expanded cervical cancer early screening and treatment options [33].

## Data Availability

Data will be available upon request from the corresponding author.

## Abbreviations

AOR: Adjusted odds ratio
CC: Cervical cancer
CI: Confidence interval
COR: Crude odds ratio
FIGO: International Federation of Gynecology and Obstetrics
Hb: Hemoglobin
TASH: Tikur Anbesa Specialized Hospital
